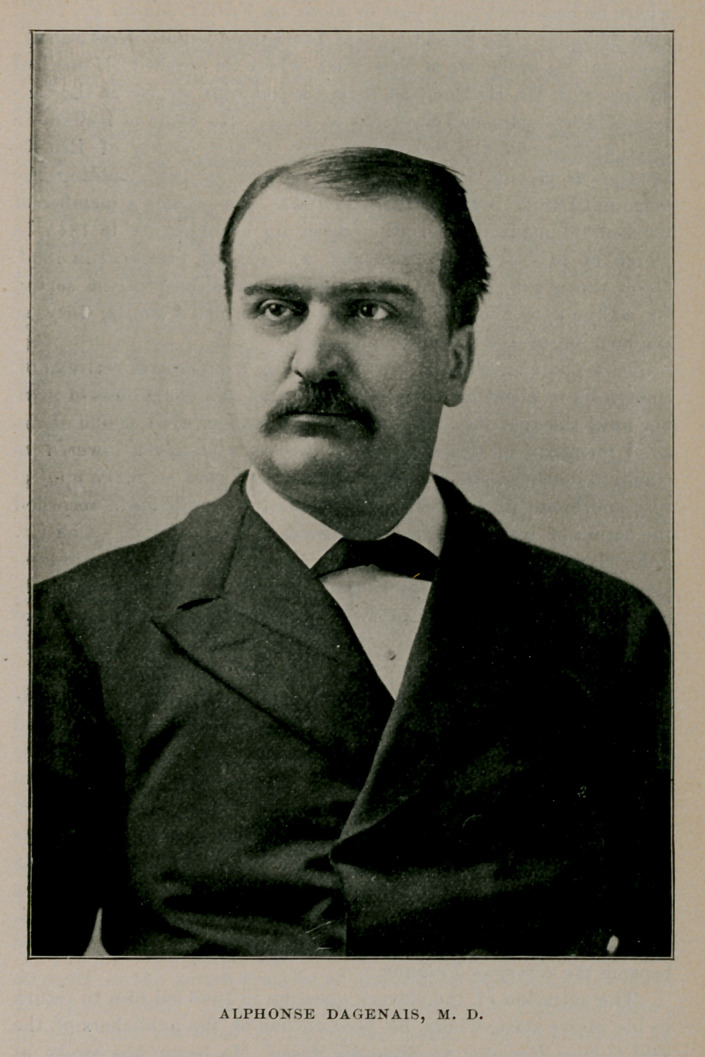# Dr. Alphonse Dagenais

**Published:** 1897-04

**Authors:** 


### Obituary.

Dr. Alphonse Dagenais, of Buffalo, died at his residence, 473 Virginia street, March 4, 1897, of apoplexy consecutive to Bright's disease, at the age of fifty years. He had been confined to his house for about two months, though his real sickness dated back into the autumn of 1896. Dr. Dagenais was a graduate of the Montreal School of Medicine and Surgery in 1867, and a licentiate of the Medical Society of the State of New York in 1870. He was a member of the College of Physicians and Surgeons of Ontario, of the American Medical Association, of the Medical Society of the County of Erie, of the Buffalo Academy of Medicine and of the Buffalo Medical Union.

At the time of his death he had been engaged in the active pursuit of his profession in this city for twenty-seven years, and succeeded in establishing an extensive and reasonably lucrative practice. He enjoyed the confidence and esteem of a large clientèle, the respect of his friends and neighbors and the friendship of his professional colleagues. Dr. Dagenais was a man of modest demeanor, possessed a most amiable disposition and stood for everything that goes to make up moral and professional rectitude. His loss is keenly felt by numerous patients who were warmly attached to him, by his friends and neighbors whose respect and confidence he enjoyed, by his professional colleagues, and especially by a wife and daughter to whom he was tenderly devoted.

ALPHONSE DAGENAIS, M. D.

## Figures and Tables

**Figure f1:**